# The Effect of Proton Pump Inhibitors on Early Implant Failure A Retrospective Cohort Study

**DOI:** 10.3390/medicina59020402

**Published:** 2023-02-18

**Authors:** Daya Masri, Noga Retzkin, Sérgio Luís Scombatti de Souza, Gil S. Slutzkey, Nirit Tagger-Green, Sarit Naishlos, Liat Chaushu

**Affiliations:** 1 Department of Oral and Maxillofacial Surgery, School of Dentistry, Tel Aviv University, Tel Aviv 6997801, Israel; 2 Department of Periodontology and Implant Dentistry, School of Dentistry, Tel Aviv University, Tel Aviv 6997801, Israel; 3 Departamento de CTBMF e Periodontia, Faculdade de Odontologia de Ribeirão Preto-USP, Sao Paulo 14040904, Brazil; 4 Department of Pediatric Dentistry, School of Dentistry, Tel Aviv University, Tel Aviv 6997801, Israel

**Keywords:** proton pump inhibitors, early implant failure, dental implant

## Abstract

*Background and Objectives*: Maintenance of a firm and long-term stable osseointegration is the primary goal of implant dentistry. Time is used to define implant failure characteristics. Early implant failure (EIF) occurs up to one year after loading. Recent studies indicated an association between proton pump inhibitors (PPI) therapy and failure of osseointegration. The present study assessed whether the use of PPIs is a risk factor to EIF. *Materials and methods:* A retrospective cohort study including 687 patients and 2971 dental implants. The study group (PPIs users) comprised 17.3% (119) individuals and 18.7% (555) implants. The remaining cohort (82.7% (568) individuals and 81.3% (2416) implants) served as control. The information was taken from the patients’ files. The following information was collected: age, gender, physical status, systemic diseases, HbA1C values before and after implant-supported prosthesis delivery in cases of diabetes mellitus, smoking, implant location, number of implants per individual, bone augmentation, implant brand, length and width, and EIF. EIF was defined as implant removal within a period of up to 12 months from loading. *Results:* EIF in PPIs vs. non-PPIs users was 19.3% vs. 14.3% (*p =* 0.16) at patient level and 5.4% vs. 3.5% at implant level (*p =* 0.03). Univariate analysis yielded factors significantly associated with PPIs use, including older age, physical status of the American Society of Anesthesiology (ASA) 3, hypertension, hyperlipidemia, diabetes mellitus, osteoporosis, cardiovascular accident (CVA), location (anterior mandible), shorter and narrower implants, and higher number of implants per individual. Multivariate analysis yielded statistically significant OR of 1.91; *p* = 0.01 for EIF following PPIs use and 2.3; *p* < 0.001 for location in anterior mandible. *Conclusions:* Patients and their healthcare providers are advised to carefully consider the potential risks of taking PPIs prior to dental implant surgery. Further research is needed to confirm these risks and elucidate systemic and local factors that may be involved in such outcomes.

## 1. Introduction

Dental implants offer a predictable and effective solution for the treatment of total or partial edentulism. Osseointegration is a direct structural and functional connection between bone and the surface of a load-carrying implant [1]. Stable osseointegration is the primary goal of implant dentistry. The process begins with the absorption of ions, proteins, polysaccharides, and proteoglycans by the Ti-oxide layer. Afterwards, macrophages, neutrophils, and osteoprogenitor cells migrate to the bone–implant interface, leading to bone apposition in close contact with the implant surface [2]. The process begins immediately with the formation of the blood clot when the implant is inserted and lasts for several months after implant insertion [3].

Dental implant success is defined using several criteria, e.g., absence of mobility; no fracture or crack; radiographic marginal bone loss <1.5 mm during the first year of function and less than 0.2 mm annually thereafter; absence of pain and/or paresthesia [4]. Dental implant survival is defined when implants are still in the patient’s jaw but criteria for success are not met [5]. Dental implant failure is a static outcome situation that requires the removal of a failed implant [6]. Despite the high survival (95% for 10 years) of dental implants, failures still occur [7].

Time is used to define implant failure characteristics. Early implant failure (EIF) occurs prior to prosthesis delivery or up to one year after loading [1]. Failure is derived from a disruption in the osseointegration healing process following, e.g., lack of primary stability or surgical trauma [6].

Late failure timing is defined as >1 year after loading [1]. Major risk factors include excessive loading [8], poor bone volume and/or quality, and existence of a chronic of peri-implantitis [6].

Factors associated with implant failure may be further classified:

- Implant-related: the material from which the implant is made, its design, surface and more.
- Surgery-related: infection control, technique complexity, loading, and more.
- Patient-related: age, gender, habits, bruxism, implant location, bone quantity and/or quality, host response as smoking, systemic diseases, past radiotherapy treatments, intake of certain medications, etc. [3].

Among the medications, it is important to emphasize the family of proton pump inhibitors (PPIs). PPIs are used worldwide as prescribed medications [9]. PPIs are a group of medications that suppress gastric acidity by inhibiting functions of the proton pump. They are the most effective anti-acid medications for upper gastrointestinal acid-related disease, such as peptic ulcer disease, Helicobacter pylori [10], gastroesophageal reflux disease (GERD), and more [9]. PPIs irreversibly inhibit the proton pump in the acid-secreting parietal cells of the stomach and thereby suppress the gastric acidity [1]. In many countries, they are available as over-the-counter medications due to their efficacy and known safety profile [9].

In 2017, new guidelines were issued to reduce the administration of the drug due to the side effects [11,12,13,14]. It seems that the reduction in the dose of the drug was superior to the replacement of the drug due to various side effects of the new drug [14].

In line with the above, and since the use of PPIs is so widespread worldwide, it is of great importance to further investigate the relationship between PPIs and dental implant failure, to reach an unequivocal decision on the issue. The present study assessed whether the use of PPIs is a risk factor to EIF. The null hypothesis was that the use of PPIs increases the chance of EIF.

## 2. Materials and Methods

The present retrospective, cohort study is based on dental records of the Department of Oral and Maxillofacial Surgery, Rabin Medical Center, Campus Beillinson, Israel. All consecutive individuals undergoing implant insertion between 2011 and 2019 were included.

### 2.1. Inclusion Criteria

- Patients following implant insertion at Oral & Maxillofacial Rabin Medical Center, Beilinson Campus, Petah Tikva, Israel
- Follow-up of at least 12 months from the date of implant loading
- Available X-rays (panoramic, computerized tomography—CT), clinical examination, and follow-up

### 2.2. Exclusion Criteria

- Follow-up < one year from implant loading.
- Bisphosphonates use
- Pregnant women
- Use of immunosuppressants
- S/P Oral and/or maxillofacial trauma
- S/P chemotherapy
- S/P head and neck radiation

The cohort included 687 patients and 2971 dental implants. The study group (PPIs users) comprised 17.3% (119) individuals and 18.7% (555) implants. Only subjects who continuatively used one of the PPI (ATC code A02BC, i.e., omeprazole, pantoprazole, lansoprazole, dexlansoprazole, esomeprazole, rabeprazole, dexrabeprazole, or a combination of these)) for at least 1 year were included in the study group.

The remaining cohort (82.7% (568) individuals and 81.3% (2416) implants) served as control.

All the implants used were two-p, iece, internal hex, rough surface titanium (Tapered ^®^ Screw-Vent Implant System, Zimmer Dental, (Warsaw, IN, USA); Lance^®^, MIS, (Bar Lev Industrial Park BAR-LEV, 2015600 Israel); MPI^®^, Ditron Dental, 2 Haofe St. South ind. Zone P.O.B 5010 Ashkelon 7815001 Israel). All treatments were performed by experienced oral and maxillofacial surgeons and prosthodontists. The study protocol was approved by the ethics committee of the Rabin Medical Center, Campus Beilinson, Israel (0674-19rmc). The present script complies with the STROBE guidelines [15]. Dental records of all individuals included were extracted and manually screened twice by 2 examiners (DM and LC).

The following information was collected: age, gender, physical status, systemic diseases, HbA1C values before and after implant-supported prosthesis delivery in cases of diabetes mellitus, smoking, implant location, number of implants per individual, bone augmentation, implant brand, length and width, and EIF.

EIF was defined as implant removal within a period of up to 12 months from loading.

### 2.3. Statistical Analysis:

The data were analyzed using SPSS software version 25 (IBM, 1 New Orchard RoadArmonk, New York 10504-1722 United States). Descriptive statistics were performed using means and standard deviations for the continuous variables and frequencies for the discrete variables. Univariate correlations were performed using the chi-square ($\chi^{2}$) test. Tests between independent samples were performed using the Mann–Whitney test. Significance was considered for a *p*-value lower than 5%.

## 3. Results

### 3.1. Univariate Analysis

To evaluate the differences between PPI users vs. non-users, univariate tests were conducted at the implant level (Table 1) and at the patient level (Table 2).

#### Implant Level

Age: As can be seen from Table 1, a significant difference was found ($\chi^{2}\left(2\right)=60.53,\;p<0.001$). Patients at the age of ≤65 are more likely not to use PPIs (60.9% vs. 43.8%). However, between the ages of 66 and 79.9 and for those over the age of 80, the likelihood of using PPIs (42.7% vs. 32% and 13.5% vs. 7.1% respectively) was higher. It can be stated that PPI users were older.

Physical status: A significant difference was found for ASA group ($\chi^{2}\left(2\right)=288.42,p<0.001$). ASA 1 individuals were more likely not to use PPIs (31.4% vs. 9.5%). Similarly, ASA 2 (42.4% vs. 27.6%). However, ASA 3 individuals were more likely to use PPIs (62.9% vs. 26.2%). It can be stated that the physical status of PPI users was significantly worse.

Systemic diseases: PPI users had significantly more systemic diseases—HTN (51.5% vs. 23.8%) ($\chi^{2}\left(1\right)=168.64,p<0.001$); Hyperlipidemia (39.6% vs. 19.9%) ($\chi^{2}\left(1\right)=97.97,p<0.001$); Diabetes Mellitus (20.9% vs.13.6%) ($\chi^{2}\left(1\right)=19.05,p<0.001$); Higher levels of HbA1c were noted for those not using PPI (7.38±1.29) vs. (6.86±0.96) (*p* = 0.001); Osteoporosis (19.6% vs. 7%) ($\chi^{2}\left(1\right)=84.25,p<0.001$); CVA (6.3% vs. 4.3%) ($\chi^{2}\left(1\right)=4.25,p=0.04)$.

Implant location: The anterior mandible was more frequent in PPI users (16.2% vs. 13%) ($\chi^{2}\left(1\right)=3.87,p=0.049$).

Implant length: Shorter implants were placed in PPI users (11.27±1.59) vs. (11.41±1.60) (*p* = 0.008).

Implant diameter: Narrower implants were used for PPI users (3.83±0.33) vs. (3.85±0.43) (*p* < 0.001).

EIF: More likely to occur in those using PPI (5.4% vs. 3.5%) ($\chi^{2}\left(1\right)=4.55,p=0.03$).

### 3.2. Patient’s Level

Age: Difference was found for age groups ($\chi^{2}\left(2\right)=15.0,p<0.001$), as those lower than 65 were more likely not to use PPI (61.1% vs. 44.5%). Yet, those between the ages of 66 and 79.9 and those over 80 were more likely to use PPI (38.7% and 16.8%, respectively) vs. (31.2% and 7.7%, respectively). It can be stated that the physical status of PPI users was significantly worse.

Physical status: Additionally, differences were found for physical status ($\chi^{2}\left(2\right)=70.66,p<0.001$). ASA 1 and 2 were more likely not to use PPI (47.6% and 36.7%, respectively) vs. (7.7% and 29.1%, respectively). However, those of ASA 3 were more likely to use PPI (63.2% vs. 25.7%). It can be stated that the physical status of PPI users was significantly worse.

Total implant number per individual: Significantly higher in PPI users (4.69 ± 3.72) vs. (4.25 ± 3.48) (*p* = 0.02). For full model tests, see Table 1 and Table 2.

### 3.3. Multivariate Analysis

A logistic regression model at the implant level showed that the independent variables significantly predict failure (χ²(13) = 27.49, *p* = 0.01), while explaining about 3% of total variance in failure. The model is well-fitted to the data (χ²(8) = 8.11, *p* = 0.42) while classifying about 96.2% of the total observations.

It was found that implants in the anterior mandible predict higher probability for EIF in comparison with other locations (OR = 2.28, *p* < 0.001). Moreover, the odds of EIF in PPI users are 1.91 times more (OR = 1.91, *p* = 0.01). For complete regression coefficients, see Table 3.

A logistic regression model at the patient level showed that the independent variables significantly predict failure (χ²(7) = 17.06, *p* = 0.02), while explaining about 4.3% of total variance in failure. The model does not fit to the data well (χ²(8) = 13.90, *p* = 0.04) while classifying about 85.1% of the total observations.

It was found that the odds of implant failure when ASA is 3 are lower than when ASA is 1 (OR = 0.34, *p* = 0.01). Yet, no difference in odds of failure was found for PPI users (*p* = 0.11) while controlling for the other variables. For complete regression coefficients, see Table 4.

## 4. Discussion

The purpose of the present study was to assess the effect of PPI on EIF. The null hypothesis was that the use of PPIs might increase the chance of EIF. Differences regarding potential modifying factors, such as age, gender, physical status, systemic diseases, smoking, implant location, number of implants per individual, bone augmentation, implant length, and width, were also evaluated.

At the implant level, the use of PPI significantly increases the chances of EIF. This can be attributed to a variety of modifying factors, such as older age, worse physical status, increased incidence of various systemic diseases (hypertension, hyperlipidemia, diabetes mellitus, osteoporosis, CVA), location in the anterior mandible, shorter and narrower implants, or higher number of implants per individual. Other modifiers, such as gender, smoking, and bone augmentation, were similar between the two groups. The logistic regression demonstrated a significant contribution only for implant location—anterior mandible (OR = 2.28).

All consecutive individuals undergoing implant insertion between 2011–2019 were included. The cohort included 687 patients and 2971 dental implants. The study group (PPIs users) comprised 17.3% (119) individuals and 18.7% (555) implants. The remaining cohort (82.7% (568) individuals and 81.3% (2416) implants) served as control. This explains the differences between the study and control group. The fraction of the study group resembles the fraction of PPI users in the population attending implant dentistry.

A recent systematic review and meta-analysis concluded that the usage of PPI has a detrimental effect on the success of dental implants. This influence needs further justification [16]. The results of the present model agree with those findings. The model found that the odds of implant failure for PPI users were 1.91 times higher, with a *p*-value of 0.01.

The results of the present study are also consistent with previously reported findings [3,16,17], which revealed a statistically significant difference in implant failure rates between PPI users and nonusers. Wu et al. [17] reported that PPIs increase the risk of failure for osseointegrated implants (133 implants in 58 PPI users). Similarly, Chrcanovic et al. [3] evaluated 999 patients (3559 implants) and reported a 12% vs. 4.5% overall failure rate for PPI users. Altay et al. [1] reported negative effect on EIF in PPI users. Conversely, Corbella et al. [18] reported that the use of PPIs, appeared to have no influence on implant survival.

Studies have indicated that using PPI might increase implant failure [1,2,3,9]. PPIs may affect bone metabolism negatively by several mechanisms including impairment of calcium absorption in the duodenum [1,3,9,10], interruption in osteoclast function in bone remolding and repair [1,9,10], a decreased overall number of osteoclasts, a decrease in bone mineral density (BMD) [1,9,10], and a decrease in granulocytes number and activity [10]. The most prominent hypothesis assumes that the reduced acidity in the stomach impairs the intestinal absorption of dietary calcium. Thus, there can be a decreased calcium absorption under PPI therapy. A recent study observed that postprandial calcium concentrations did not increase in subjects on a PPI regime, whereas control subjects demonstrated a clear spike in serum calcium levels. They also observed that the urine calcium excretion in the PPI group was reduced in comparison to the control group [3,11,12].

In a study performed on mice, it was found that genetically manipulated mice were achlorhydric (with absence of hydrochloric acid from gastric juice), presented decreased serum calcium levels, and developed osteoporosis and secondary hyperparathyroidism to maintain calcium balance [3], leading to a lower degree of BMD [1]. On the other hand, in a study performed on laboratory rats that had an implant inserted in the tibia bone, it was reported that the calcium concentration in the tibial bone defects were not altered after two weeks of exposure to omeprazole. These discrepancies could be attributed to differences in the dose and the duration of treatment between the two studies [10].

Osteoclastic cells play a cardinal role in the early and later stages of bone healing process. PPIs are also known to down-regulate the osteoclasts activity by increasing the expression of osteocalcin and osteoprotegerin (OPG). This is how the activation levels of Receptor Activator of Nuclear Factor Kappa-B Ligand (RANKL) is affected [10,13].

It is also possible that PPIs may promote decreased bone turnover by preventing the V-ATPase of osteoclasts, similar to the way PPIs inhibit gastric hydrogen potassium ATPase, however at higher levels [13], which has a direct negative effect on bone cells [1,13].

Another effect of PPIs on bone metabolism is the decrease in osteoclastic differentiation mediated by osteoblastic cells [1,13]. A study performed on the tibia of rats showed a significant reduction in the total number of osteoclasts in the bone defects of rats exposed to omeprazole compared to the control group. The researchers explained that this probably occurred because PPIs inhibit osteoclasts bone resorption and decrease the expression of certain genes associated with osteoclastic activity and differentiation [10]. Further findings support the hypothesis of a possible direct effect of PPIs on bone mineral metabolism [9]. Although many epidemiological studies reported that PPI treatment reduces BMD, others failed to find a significant association. Moreover, after a broad literature review, no significant difference was found in the mean values of BMD between PPIs users and controls [9,12]. Since PPIs inhibit the granulocytes number and function, in theory, PPIs could also affect bone healing and osseointegration through suppression of granulocytes [10]. A study shows that the administration of omeprazole did not affect the number of granulocytes. Granulocytes accumulate in the fracture callus as part of the inflammatory response of the early stages of bone repair. These cells release angiogenic factors and proteases that promote formation of new blood vessels and degradation of the soft callus. As noted, no damage to the granulocytes was found under the influence of omeprazole. However, we could not rule out the effect on granulocyte function since PPIs can inhibit the function of granulocyte without affecting their numbers. Therefore, the effect of PPIs on granulocytes function in bone healing should be assessed in future studies [10].

A recent histomorphometry study in dogs assessed the effect of systemic administration of omeprazole on osseointegration around titanium dental implants. They concluded that systemic administration of PPIs may interfere with osseointegration of dental implants [19].

A recent study found no independent associations between PPI use and implant failure or peri-implantitis. They concluded that, contrary to published literature, PPIs may not influence implant health [20].

The strengths of the present study lie in the large number of PPI users (119 individuals) and implants (555). All implants were inserted by experienced well trained maxillofacial surgeons in a single academic center. The entire cohort is also large, with 687 individuals and 2971 implants. A large number of modifying factors was also considered. However, the findings should be interpreted with caution, and further research is needed to confirm these results. Additionally, it is possible that the relationship between taking PPIs and implant failure may vary depending on other factors that were not accounted for in this study, such as the specific type of PPI being used or the duration of PPI use. A recent review [21] concluded that PPIs represent a risk factor for dental implant survival. However, limited research is available. Hence, prospective randomized controlled trials should be carried out to elucidate the effect of PPI on osseointegration.

## 5. Conclusions

PPI use may increase EIF (OR = 1.91; *p =* 0.01). Until more is known about this relationship, it is important for patients and their healthcare providers to carefully consider the potential risks and benefits of taking PPIs after dental implant surgery and to make decisions based on the best available evidence.

## Figures and Tables

**Table 1:** Univariate tests at the implant level.

		PPI Non Users		PPI Users		
Variable	Group	*N* (%)	M±SD	*N* (%)	M±SD	*p*-Value
Age Groups (Years)	≤65	1472 (60.9)		243 (43.8)		<0.001
	66–79.9	772 (32)		237 (42.7)		
	≥80	172 (7.1)		75 (13.5)		
Physical Status	ASA 1	759 (31.4)		53 (9.5)		<0.001
	ASA 2	1025 (42.4)		153 (27.6)		
	ASA 3	632 (26.2)		349 (62.9)		
Systemic Diseases						
Hypertension	Yes	575 (23.8)		286 (51.5)		<0.001
Hyperlipidemia	Yes	480 (19.9)		220 (39.6)		<0.001
Diabetes Mellitus	Yes	328 (13.6)		116 (20.9)		<0.001
HbA1c before			7.38 (1.29)		6.86 (0.96)	0.001
HbA1c after			6.84 (1.18)		6.49 (0.88)	0.13
Delta HbA1c			0.54 (0.79)		0.36 (0.74)	0.07
Osteoporosis	Yes	170 (7.0)		109 (19.6)		<0.001
CVA	Yes	103 (4.3)		35 (6.3)		0.04
Smoking	Yes	155 (6.4)		40 (7.2)		0.50
	No	2261 (93.6)		515 (92.8)		
Augmentation	Pristine	920 (38.1)		233 (42)		0.09
	Augmented	1496 (61.9)		322 (58)		
Implant Location						
Anterior maxilla	Yes	376 (15.6)		79 (14.2)		0.43
Premolar maxilla	Yes	458 (19.0)		90 (16.2)		0.13
Posterior maxilla	Yes	371 (15.4)		74 (13.3)		0.23
Anterior mandible	Yes	315 (13)		90 (16.2)		0.05
Premolar mandible	Yes	402 (16.6)		103 (18.6)		0.28
Posterior mandible	Yes	492 (20.4)		114 (20.5)		0.93
Implant Parameters						
Length			11.41(1.60)		11.27 (1.59)	0.008
Width			3.85 (0.43)		3.83 (0.33)	<0.001
EIF	Yes	84 (3.5)		30(5.4)		0.03

**Table 2:** Univariate tests at the patient level.

		No PPI		PPI		
Variable	Group	*N* (%)	M±SD	*N* (%)	M±SD	*p*-Value
Gender	Female	344 (60.7)		79 (66.4)		0.24
	Male	223 (39.3)		40 (33.6)		
Age groups (years)	≤65	347 (61.1)		53 (44.5)		0.001
	66–79.9	177 (31.2)		46 (38.7)		
	≥80	44 (7.7)		20 (16.8)		
Physical status	ASA 1	213 (37.6)		9 (7.7)		<0.001
	ASA 2	208 (36.7)		34 (29.1)		
	ASA 3	146 (25.7)		74 (63.2)		
Augmentation	Yes	316 (55.6)		67 (56.3)		0.89
Smoking	Yes	29 (5.1)		8 (6.7)		0.48
Number of implants per individual			4.25 (3.72)		4.69 (3.48)	0.02
EIF	Yes	81 (14.3)		23 (19.3)		0.16

**Table 3:** Binary Logistic regression coefficients (at the implant level) to predict implant failure.

	OR	95% CI Lower	95% CI Upper	*p*-Value
Age group (≤65)	0.99	0.65	1.51	0.97
Physical status (ASA 2)	0.60	0.36	1.01	0.06
Physical status (ASA 3)	0.65	0.36	1.15	0.14
Hypertension	0.77	0.45	1.33	0.35
Hyperlipidemia	0.93	0.54	1.62	0.81
Diabetes Mellitus	0.94	0.50	1.75	0.84
Osteoporosis	0.89	0.42	1.88	0.76
CVA	1.03	0.40	2.65	0.95
Anterior mandible	2.28	1.46	3.56	0.00
Implant length	1.09	0.96	1.24	0.18
Implant width	1.13	0.70	1.80	0.62
PPIs use	1.91	1.19	3.08	0.01

Note: The reference group for the age group variable is “above 65”. The reference group for ASA is “ASA 1”.

**Table 4:** Binary Logistic regression coefficients (at the patient level) to predict implant failure.

	OR	95% CI Lower	95% CI Upper	*p*
Age group ≤ 65	1.03	0.64	1.67	0.89
Physical status (ASA 2)	0.69	0.41	1.16	0.16
Physical status (ASA 3)	0.34	0.15	0.78	0.01
Number of implants per individual	1.05	1.00	1.11	0.07
PPIs use	1.61	0.90	2.87	0.11

Note: The reference group for the age group variable is “>65”. The reference group for ASA is “ASA 1”.

## Data Availability

The datasets generated during and/or analyzed during the current study are available from the corresponding author on reasonable request.
